# The Role of Institutional Engagement at the Macro Level in Pushing the Circular Economy in Spain and Its Regions

**DOI:** 10.3390/ijerph17062086

**Published:** 2020-03-21

**Authors:** María del Mar Alonso-Almeida, José Miguel Rodríguez-Antón

**Affiliations:** Business Administration Department, Universidad Autónoma de Madrid, 28049 Madrid, Spain; mar.alonso@uam.es

**Keywords:** circular economy, institutional pressure, mimetic pressure, coercive pressure, normative pressure

## Abstract

Currently, the European Union is promoting the circular economy, a change that involves moving the foundations of actual economies toward the most sustainable production and consumption periods, in which the reuse of resources predominates, mainly through recycling, reuse, and reduction, among other strategies. This study, through the application of institutional theory, analyzes the role that institutional pressure has in the diffusion and adoption of the circular economy from the state to the regions inside through coercive, normative, and mimetic pressures. A matrix of institutional positioning was developed that analyzes the number and diversity of circular economy initiatives. The results show that coercive pressure followed by mimetic pressure are the most relevant in explaining the development of the circular economy in Spain in relation to the closest other European countries in Southern Europe, while there is low normative pressure. The results obtained provide relevant information on how to accelerate the development of the circular economy throughout the European Union through the adequate exercise of different types of institutional pressure.

## 1. Introduction

There is no doubt that the circular economy (CE) is becoming a new economic model that will gradually displace the traditional model of linear economy based on the extraction of raw materials, manufacturing—with the necessary use of energy factors, consumption of goods produced, and disposal of materials that have become obsolete or that have lost properties or capacity for use. This topic is quite recent; the first article was published in 2006. However, in 2015 this topic started to acquire importance in academy, economics, and social issues [1,2]. In practice, the circular economy (CE) is being strongly promoted in countries, such as China, and economic areas, like the European Union (EU), to promote economic growth and sustainable environmental development [3]. Nevertheless, in Europe the research is primarily focused on Northern and Central Europe with very scarce research on Southern Europe [1].

The approach the EU uses to deploy its directives, policies, and recommendations is a “top-down” transformation, which means that that they first change national regulations and then other lower regulations that could be affected. Therefore, it is important to know the situation and development in Spain for two main reasons: (1) because it can serve as a model for implementation and development of the CE to be followed by other southern European countries, given their particular composition of regional areas that have partially transferred the capacity to legislate on certain areas and function as independent states with respect to principal states; and (2) for the importance in Spain of sectors such as tourism, agriculture, construction, livestock, or the automotive industry, which are a true reflection of the EU as a whole. In addition, the importance of the tourism sector in southern European countries is unquestionable. All of them are in the TOP 50 of the world ranking for tourism competitiveness (Spain, 1st; France, 2nd; Italy, 8th; Portugal, 12th; Greece, 25th; Croatia, 27th; Malta, 35th; Slovenia, 36th, and Cyprus 44th) and in the TOP 20 of the EU27 ranking (Spain, 1st; France, 2nd; Italy, 4th; Portugal, 6th; Greece, 12th; Croatia, 14th; Malta 16th; Slovenia, 17th, and Cyprus, 20th) [4].

Under these premises, the research question that this paper attempts to answer concerns the role of institutional theory in pushing the CE through the role of the state, specifically Spain, in the other territories over which it exerts influence, its regions. To do this, it will be analyzed (1) how European legislation related to the implementation of the CE has evolved; (2) what the position of Spain is in the implementation of the CE using the official information offered by Eurostat through the monitoring framework; (3) what the development of the CE in Spain is and the type of institutional pressure that is being developed; and (4) what role the Spanish State is having in the promotion of the CE in the different regions of the country, analyzing the threats and opportunities existing for the achievement of the objectives set and the development of the CE in the European Union (EU).

Previous literature has highlighted the role that institutional pressure has in promoting behavior at the macro and micro levels. In fact, it emphasizes the value that institutional theory provides a valuable alternative lens to other theories and explains possible future pathways [5] and puts institutional theory above other theoretical bases to explain the changes that occur in the environment [6]. However, this theory has usually been applied to companies and their behavior in the face of environmental pressures to adopt a certain practice or management system [7] and not to territories or regions. Therefore, this exploratory research aims to apply institutional theory to a specific policy, the CE, and its adoption in a certain territory and its regions. This is mainly for two reasons: First, the CE is in its infancy, and there is still no clear explanation for its adoption and diffusion at the macro level; second, it is necessary to know which pushers are the most important in its adoption at that level.

In addition, this research makes several contributions to the literature on the topic. First, the relationship between institutional pressures and the circular economy have not yet been analyzed, according to our best knowledge. However, high level institutions have the power to formulate rules and reward compliance or sanction noncompliance to other organizations based on their superior position and legitimacy [8,9]. Second, this study explores the dimensions of institutional pressure—coercive pressures, normative pressures and mimetic pressures—and their influence on the circular economy in geographical areas, which enriches the literature on the preconditions of the circular economy and offers a holistic view of the drivers of the circular economy that prior studies have failed to obtain. Coercive and normative pressure have a significant positive impact on changing the behavior of an environment, but the role of mimetic pressure is uncertainly [10,11,12]. Third, this study, situated in UE, which is formed by territories with distinct political and economic characteristics, is of significant importance to testing how circular economy could be adopted homogeneously by the most of territories.

To achieve these goals, this paper is organized as follows. Section 2 presents an analysis of how the legislation of the EU has evolved from the rational use of resources to the implementation of a specific model of CE. Section 3 presents a review of the literature on institutional theory and CE. Section 4 introduces the EU CE monitoring framework and its results. Section 5 shows the applied research method. Section 6 presents the results and discussion, and the paper is finalized with the presentation of conclusions and limitations and directions for future research.

## 2. Legislative Analysis: EU Legislation on the Circular Economy

The EU is focused on engaging resources to make the CE a reality. European Commission [13] indicates that in the circular economy “… the value of products, materials, and resources is maintained in the economy for as long as possible, and the generation of waste minimized, …”

In order to measure the degree of progress towards the CE in the EU, the European Commission [14] presents a set of indicators, which will later be analyzed Table 1 shows the legislative development of the CE in the EU. In this Table, all the Communications, Directives and Reflections issued by the European Commission, which reflect the role of institutional engagement at the macro level in pushing the circular economy, have been collected from 2011 to the present. Most of them are Communications that seek to establish a framework for action by the member countries of the EU to guide their actions towards the desired CE.

It is vital to analyze the impact of this legislative impulse in order to understand if the objectives pursued are being achieved. Therefore, the literature that relates institutional theory to the adoption of environmental practices is reviewed below.

## 3. Institutional Theory and the Adoption of Environmental Practices

Currently, the protection of the environment has been influenced and supervised by governments at all levels [15]. Sometimes, governments have taken the lead in protecting natural resources and the environment, but, in other cases, governments have acted when pushed by international organizations, and it is true that governments have impacted the behavior of businesses to protect the environment [16].

Analyzing CE from the viewpoint of institutional theory, CE could be extended due to external pressure, especially from governments [16]. According to institutional theory, institutions are organizations they have the power to formulate rules and reward compliance or sanction noncompliance to other organizations based on their superior position and legitimation [8,9]. In the context of governments, higher level governmental institutions are in a position to promulgate that type of mandatory laws for lower position governments and eject a direct pressure over them. Obviously, these institutions not only provide mandatory rules but are also in a position to guide and give recommendations or other resources to help lower position organizations [17].

In the literature, Zeng et al. [18] asserted that institutional pressure has a significant impact through three lines of action: (a) Coercive pressure is exerted by laws and based on a system of rules, sanctions, and rewards [19]; (b) normative pressure is promoted by regulations, recommendations, and rules provided to reach certain goal [17]; and (c) mimetic pressure is exerted by imitating behavior that others have perceived as similar. This type of pressure arises when there is a high level of uncertainty on how to solve a specific problem, perform a specific activity or reach a specific goal [9]. In the specific case of governments, governments in the same level (national, regional, or local) could pursue mimetic behavior.

Thus, according to Zeng et al. [18], the first type of pressure is considered to be a hard driver, pushing certain situations, and the second, a soft driver. The latter is a type of indirect pressure by one government based on other governments in the same level of behavior. Usually, these influences could converge to achieve the same goals [20]. In addition, different institutional pressure types exert different degrees of pressure [21]. Thus, coercive pressure has a higher degree of pressure to adopt or extend a certain issue. Zhu et al. [10] found that coercive pressure was more likely to lead to the adoption of green procurement and recycling policies in companies. In this line, Simpson [11] found that a number of countries promulgated laws on recycling when the European law was effective in reducing waste. Further research found the same or that coercive pressure from governments has a significant positive impact in changing the environment and behaviors (e.g., [12,22,23] or a positive impact [24]). Thus, coercive pressure could be the first driver to push CE. Therefore, an active role of central governments seems to be essential to promote a certain behavior such as CE development. On the contrary, a central government’s passive role could act as an inhibitor to the development of a CE, due to CE development requiring certain positive environmental conditions [25]. Therefore, it will be expected that coercive pressure was the main driver to push the CE among countries and regional governments. Thus, the following proposition is proposed:

**Proposition** **1:** *Coercive pressure has a highly positive influence on pushing CE development from a state to its regions*.

Normative pressure also provides a framework to push certain issues [20]. Normative pressure in the context of governmental context could be determined by recommendations, guides, or plans from the central government to other lower level governments. Thus, normative pressure could drive the adoption of the CE in such regions. Ranta el al. [26] analyzed institutional drivers and barriers to the CE in recycling efforts in China, Asia, and Europe. They found that regulatory measures increase recycling. However, the adoption of certain behaviors could be slower than in the case of coercive pressure. Specific plans for regulation require the engagement of a variety of stakeholders in order to achieve advances. Therefore, regulation can help to push the CE and make it faster or slower depending on the position of key players. However, most previous research has shown a positive influence in changing environmental behavior [12,22,23,24]. Nevertheless, institutional pressure could also inhibit deployment of the CE when the normative system is misaligned with the goals of the CE or when the implementation implies costs and there is no support for it [7]. Institutional support could be a source of crucial knowledge, advice, and funding to implement the CE in regions [27]. Accordingly, it will be expected that normative pressure was a relevant driver to push the CE among countries and regional governments. Therefore, the following proposition is proposed:

**Proposition** **2:** *Normative pressure has a positive influence on pushing CE development from a state to its regions*.

Finally, mimetic pressure can be an enabler for the CE when coercive or normative pressures fail. Thus, regional governments could follow another government’s behavior when they observe some advantages in adopting such behavior. Thus, this type of pressure is voluntary and could be considered self-imposed [20]. Usually, it is useful to minimize the risk to push something new or an uncertain situation [7]. Mimetic pressure runs under environmental uncertainty, ambiguity, and complexity in order to reduce them [27].

Haunschild and Miner [28] identified three sources of mimetic imitation. These sources were frequency, trait, and outcome based. Frequency basis is produced when organizations adopt a certain behavior without thinking, only motivated because all equals are doing it. Trait basis is produced by identification with selected traits such as size, performance, or proximity. Finally, outcome-based imitation is the result of observing the outcomes produced. Thus, leaders or the best-in-class are the main source of imitation [6]. Therefore, regions could look to imitate the state positioning in frequency based imitation. On the other hand, they could also mimic due to some elements of identification, such as size in population or surface, proximity, or percentage gross domestic product (GDP) input. Nevertheless, outcome based imitation is less probable because CE outcomes in regions are very low or still non-existent.

Mimetic pressure seems be less powerful than coercive and normative pressures, but it can run when a government neglects or delays adoption of the CE. Nevertheless, mimetic pressure is weak when there is a new phenomenon and there is little information about how to adopt and develop certain behaviors or the outcomes such as the CE [29]. In addition, mimetic pressure is low when a society is not educated enough regarding why sustainability is important and how the CE can be beneficial for it.

Research regarding mimetic pressure results have shown mixed results. Thus, Wu et al. [24] found negative and no significant effects in a green supply chain adoption. However, innovative institutions can be the first ones to be imitated [27]. The main reason is that they are considered a source of information to reduce uncertainty [20]. Therefore, it will be expected that mimetic pressure can also be pushed by regional identification although in less measurement that coercive and normative pressures. Thus, the following proposition is suggested:

**Proposition** **3:** *Mimetic pressure has a less positive influence on pushing the CE among regions*.

## 4. EU CE Monitoring Framework

On January 16, 2018, the European Commission published a communication entitled “Communication from the Commission to the European Parliament, the Council, the European Economic and Social Committee and the Committee of the Regions on a monitoring framework for the circular economy” [30], which provides an analysis of how member states are implementing the measures aimed at implementing a CE in the EU and what results are being achieved, with the objective of determining whether these measures are sufficient to achieve the intended objective and, where appropriate, what measures should be strengthened or what new measures could be adopted.

The monitoring framework offers a set of basic indicators of the main elements that make up the CE, including the lifecycle of products and materials, the priority areas and sectors, and the impacts on competitiveness, innovation, and jobs. In addition to offering specific information on these indicators, it shows the trend or evolution that they have been experiencing in recent years and identifies the best practices implemented by member states so that they can be disseminated. The framework is collected on the Eurostat website [31] and is updated as countries provide specific data on the indicators considered. The monitoring framework has some indicators grouped into four aspects of the CE: (1) Production and consumption—EU self-sufficiency for raw materials, green public procurement, waste generation, and food waste; (2) waste management—overall recycling rates and recycling rates for specific waste streams; (3) secondary raw materials—contribution of recycled materials to raw material demand and trade in recyclable raw materials, and (4) competitiveness and innovation—private investments, jobs, gross value added, and patents.

Although these indicators can offer a general idea about the degree of implementation of the EC in Europe, its relationship with other variables, such as the GDP of each country, its sectorial structure, the structure of its balance of payments, etc., could be relevant. Anyway, we have calculated the correlation coefficient between the values of municipal waste generation and the 2018 GDP of the nine countries of Southern Europe on which we have conducted the study, and the value obtained is R^2^ = 0.14289735, so we rule out that the country’s GDP significantly determines the level of municipal waste generation per capita.

This monitoring framework [30], indicates that in the EU in 2014, “8 billion tons of materials are processed into energy or products,” but “only 0.6 billion tons originate from recycling.” Furthermore, “out of the 2.2 billion tons of waste that are generated only 0.6 billion tons re-enter the system as recycled materials. The rest of the materials is waste.” In addition, in 2016, only 36.4% of the raw materials used in the EU come from its member countries; that is, it is moderately self-sufficient, hence the importance of making good use of the consumption of raw materials. These data show an important potential for improvement “by increasing the share of materials recycled as secondary raw materials and decreasing the production of waste.” [30].

As can be seen in Table 2, since the year 2015, the EU has been making an important effort to favor the implementation and development of the CE. Specifically, in aspects related to production and consumption, the generation of municipal waste is slowly being reduced, going from 498 kg per capita in 2011 to 488 kg in 2018 and, with food waste, going from 81 million tons in 2012 to 80 million tons in 2016.

With regard to the effort that the EU is making in the management of waste through timely recycling, 47% of municipal waste was recycled in 2018 compared to 39.6% in 2011. Of this waste, the types that are recycled in the greatest percentages are overall packaging, which reached 67% in 2017 compared to 64.7% in 2012; electrical and electronic equipment (e-waste), which went from 28.7% in 2011 to 41.4% in 2016, and bio-waste, which reached 69 kg per capita in 2011 compared to 83 kg per capita in 2018. Finally, the recovery rate of construction and demolition waste has increased from 78% in 2010 to 89% in 2016. Regarding secondary raw materials and the end-of-life recycling input rates (EOL-RIR), the last data available was scant at 11.7% in 2017, and the circular material use rate has gone from 10.8% in 2010 to 11.7% in 2017. Regarding the competitiveness and innovation aspect, the gross investment in tangible goods, measured through the percentage of GDP at current prices, has increased slightly, going from 0.11% in 2013 to 0.12% in 2017. On the other hand, the percentage of persons employed has gone from 1.68% in 2012 to 1.69% in 2016. Finally, the number of patents related to recycling and secondary raw materials (a fraction of the patent family is allocated to each applicant and relevant technology) has gone from 0.60 per million inhabitants in 2009 to 0.70 per million in 2015.

In addition, there is also no specific area of the EU that excels in these efforts. As can be seen in Table 3, Romania is the country in the EU that generates the least municipal waste per capita, standing at 272 kg per capita in 2018 compared to the 488 kg per capita EU average. As for waste management, Germany, Belgium, Croatia, Austria, Luxembourg, The Netherlands, and Malta lead the recycling and recovery for specific waste streams—municipal waste, overall packaging, e-waste, bio-waste, and construction and demolition waste. As regards secondary raw materials, The Netherlands excels in the circulating material use rate, with an estimated value of 29.9% in 2017 compared to a scant 11.7% on average for the EU countries in that same year. Finally, regarding competitiveness and innovation, Latvia stands out; it employed, in activities related to the CE, 2.82% of total employees in 2017 compared to the 1.69% EU average of that year. Luxembourg, which in 2015 managed to generate no less than 3.51 patents per million inhabitants compared to the EU average of 0.70, is also notable.

Regarding Spain, while some indicators show that this country is making a significant effort in the CE sphere, others indicate that it is still far from the EU average values (see Table 4). Specifically, in aspects related to production and consumption, Spain generated, in 2018, a total of 475 kg of municipal waste per capita compared to the average 488 kg per capita in the EU, which indicates an important effort to control the generation of municipal waste.

Regarding the efforts made in the management of waste through timely recycling, Spain is still far from the EU average for recycling, with only 36% of municipal waste compared to the EU average of 47% in 2018. However, the percentage of recycling of overall packaging was, at 68.5% in 2017, even higher than the EU average of 67%. The same happens in the case of recycling bio-waste; Spain recycled 84 kg per capita, against the average of 83 kg per capita of the EU in 2018. On the other hand, the same does not happen with the recycling rate of electrical and electronic equipment, since Spain was below the EU average when Spain recycled 41% compared to the EU average of 41.4% in 2017, and the recovery rate of construction and demolition waste was also lower in Spain, where in it reached a rate of 79% compared to 89% for the EU in 2016.

In regards to secondary raw materials, the circular material use rate of Spain in 2017 was 7.4% compared to 11.7% in the EU, so there is still margin for improvement. In the sphere of the competitiveness and innovation aspect, in 2017, Spain only dedicated 0.10% of its GDP at current prices to the gross investment in tangible goods compared to the 0.12% of the EU. In contrast, Spain hires a greater percentage of people in the CE field of total employment than the EU, because Spain hired 2.04% of all employees compared to 1.69% of the EU. Finally, as regards the number of patents related to recycling and secondary raw materials, Spain registered, in 2015, 0.43 patents related to recycling secondary raw materials per million inhabitants in contrast to the EU average of 0.70.

In relation to the ten CE variables considered, Spain is above average in four and below average in six, which can be determined by the clear orientation that Spain has toward the services sector and especially toward the tourism sector (Spain is the second country in the world for both international tourist arrivals and for tourist income), moving away from more industrial sectors in which, traditionally, more attention has been paid to the treatment of waste (Spain is only the fourth by contribution of the industrial sector to GDP of the nine countries analyzed). Consequently, it occupies a very prominent position with respect to the countries of its geographical environment—that is, Southern Europe or Mediterranean Europe, configured by Croatia, Cyprus, France, Greece, Italy, Malta, Portugal, Slovenia, and Spain. To demonstrate this claim, a comparative study of each of these Mediterranean countries has been carried out concerning each of the selected CE indicators. Given that the measurement magnitudes are heterogeneous percentages, kg per capita, and number of patents per million inhabitants, a value of one has been granted to the country that led each indicator, a value of two to the one that remained in second position, a value of three to the third, and so on until the last one. Once these scores were granted, the scores for each country were added together, and the country that obtained the lowest total was considered the leader in application of the CE, the one with the second lowest total was considered the second best for application, and so on.

As can be seen in Table 5, Spain is the third country of the nine Mediterranean countries in terms of the application of the CE, being only behind Italy and Slovenia, which may indicate a certain leadership among the countries in this environment. In particular, this good position is achieved due to the fact that it is the one country of the nine Mediterranean countries that has the second lowest generation of municipal waste, with 475 kg per capita in 2018. Likewise, it is very well positioned, occupying the third position in terms of percentage of overall packaging recycling, reaching 68.5% in 2017, as well as in the recycling rate of e-waste, reaching a recycling percentage of 41% of those items in 2017.

## 5. Methods

### 5.1. Data of Analysis: Spain’s Positioning in the Circular Economy; Development of Policies and Norms

Following the recommendations issued by the European Commission and the directives of the European Parliament and the Council of the EU relating to the CE, Spain has assumed its commitment to the development of the CE and based on a joint function between several ministries. On September 18, 2017, The Ministry of Economy, Industry, and Competitiveness and The Ministry of Agriculture and Fisheries, Food, and Environment promoted the “Pact for a Circular Economy.” It had been subscribed to, initially, by 53 companies and institutions, and by 02/04/2020, by 357. In the next step, these two ministries exposed a public opinion with the spirit of involving all stakeholders (public, administrations, businesses, civic organizations, and citizens) on February 12, 2018—the draft of the Economy Strategy of the Circular Economy, which includes a first action plan (2018–2020) with 70 measures aimed at achieving a more efficient use of natural resources that is endowed with a budget of €836,789,110.98. Similarly, on June 15, 2018, the government of Spain published the “Action Plan for the Implementation of the 2030 Agenda. Toward a Spanish Strategy for Sustainable Development,” which shows the importance that the CE has for achieving sustainable development. However, the political instability prevailing in Spain since that year, with successive changes of government that provoke brief electoral mandates, has prevented legislative procedures from being processed and approved and the task of a CE model is encouraged at the national level. The above results are the result of the fall in the predominant economic activity in Spain—the construction sector—and the effort made during the period of the economic crisis (2007–2017), which caused changes in production and consumption processes. This situation has been used to start the transition toward a more sustainable economy [32]. In fact, although prior to 2017 the legislative roll-out regarding the CE at the state level was low, since that year, progress has been made in the monitoring framework.

### 5.2. Data Gathered and Analysis

At the beginning of 2017, the indications regarding the CE in Spain were incipient and focused on the final part of the production cycle—that is, on waste management in line with the European Waste Directive, the driver of the coercive form. That is why it is a series of measures to improve the CE in line with European directives. In particular, a national roadmap for the CE should be marked as a central base [32].

In order to analyze the situation of CE development in Spain, the following steps were addressed with governmental measurements developed since January 2017 to December 2019. This research was conducted on the methodology based on [33,34], consisting of a three-step process to revise and evaluate initiatives for CE based on regional development. The total CE initiatives in the Spanish state and each Spanish region were searched for and classified—17 regions and two cities in North Africa, and, finally, the initiatives were separated into laws and other norms. To guarantee the accuracy and quality of the process, every step was tested and controlled first by one research assistant and second by two senior researchers.

**Figure 1:**
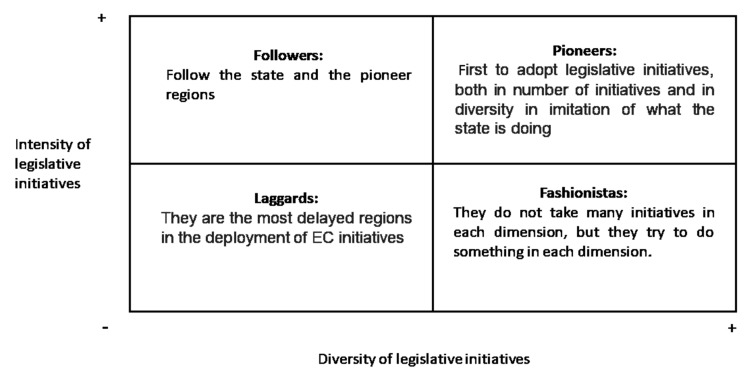
Matrix for adopting CE legislative initiatives. Source: Own elaboration based on [23,36].

To measure the CE positioning and development in regions and, based on the study by [35], to classify the different regions, a matrix was created that helps to identify both the intensity of legislative initiatives (y) and regulations generated, measured in the number of legislative initiatives, and diversity of legislation and regulations generated, measured by the number of legislated and/or regulated dimensions (x). The measure of intensity of adoption in different variables has been used by previous research as a proxy to identify the pressure exerted by the environment and the organizational response [23,36]. Thus, a pair of values (x, y) by region were calculated that summarized initiatives by dimension and type by region. In this way, we have the following matrix as shown in Figure 1. According to [35], pioneers are the first to adopt CE legislative initiatives in both axes, intensity of initiatives (y) and diversity of initiatives (x). On the other hand, laggards delay in adopting such initiatives. In the middle, there are situations where followers choose to follow the pioneers’ behavior, but with a certain delay. They are neither the first nor the last to adopt CE. Finally, fashionistas usually take more time to adopt a large number of initiatives, but they look to take advantage of any initiative of the CE dimension.

In addition, a mapping was developed by analyzing, in depth, the type of initiative in each region, we can analyze the coercive and normative pressures. To this end, the number of existing coercive initiatives in each region and the number of initiatives considered normative by each region within the framework mentioned have been identified by each of the dimensions of the CE Spanish monitoring framework. The average is then calculated in the number of initiatives—coercive or normative—for each dimension of the framework. When the number of initiatives is above average, it is considered a high intensity of development and is shown in green. If the mean is in the middle level of that dimension, the development is considered usual in the analyzed sample, and it is represented in orange. Finally, when the average of the dimension is below average, it is represented in red, indicating a deficient development of initiatives in that dimension and region.

**Figure 2:**
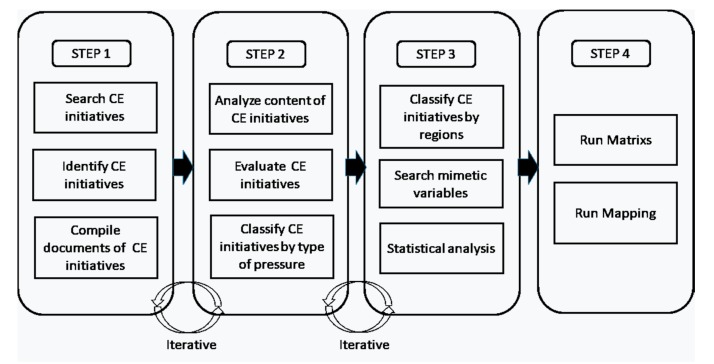
Methodology of the study. Source: Own elaboration enlarges the methodology used by [33,34].

Finally, in a four-step statistical analysis, the matrix and mapping analysis developed is shown in Figure 2.

## 6. Results and Discussion

### 6.1. CE in Spain: Results

Table 6 shows the legislative initiatives that have been developed in Spain, from 2017 to 2019, for each of the dimensions established in the monitoring framework of the EU. The total initiatives by type have been gathered and calculated.

Initiatives have been developed irregularly during the period studied (see Table 6). The largest legislative and regulatory effort occurred in 2017. The order of priority was waste management, production and consumption, secondary raw materials, and competitiveness and innovation. This order has continued in the following years. In contrast, in the EU, the order of legislative priorities was competitiveness and innovation, waste management, production and consumption, and secondary raw materials.

When the data contained in Table 6 are analyzed in depth, it can be seen that, with regard to hard regulations, the legislative initiatives deployed by the state are few compared to the total number of initiatives. These legislative initiatives are focused on waste management and production and consumption. The first case is in response to the coercive pressure of European regulations, and the second is a response to link European regulation and an impulse for a progressive change towards more circular economy. As can be seen in Table 6, the greatest effort is being made in the management of waste and its reuse as part of the main objectives of the CE directive—coercive pressure and rule—and production and composition to push a real change in the economy while the number of regulatory initiatives is extensive, encompassing plans, strategies, and programs. The legislative initiatives consist of concrete proposals and plans related to the competences of the state, which also serve as a basis for the initiatives that regional governments should take within the scope of their competences. On the other hand, most of regulatory initiatives have been submitted to public consultation in a way that involves the main stakeholders from each of the initiatives. This process of co-production and collaboration provides a number of advantages, such as mutual understanding and learning; stakeholder engagement; innovation in the way to awareness of the topic; including different types of knowledge, and increasing credibility and trust [37].

Nevertheless, initiatives in secondary raw materials and competitiveness and innovation dimensions are scarce. It seems to be a contradiction, because previous research has asserted that radical changes in the market, such as CE, need innovation [38] and specific plans to reuse raw materials [39]. Thus, the pushes in production are not correlated with other critical dimensions.

Therefore, we will now analyze the positioning of each of the regions that make up the Spanish State.

### 6.2. Regional Contributions to the CE in Spain: Results

Institutional theory provides a framework that helps to understand how organizations move from a particular initial position to a new position with respect to a particular practice [6,40], specifically the CE. Given the legislative effort made during 2017, the regional areas, following in the wake of the state, have developed different political initiatives deployed at a coercive level in the form of laws and regulations. When analyzing the CE initiatives in each of the regions, according to the Spanish monitoring framework, as previously mentioned, different positions were found.

**Figure 3:**
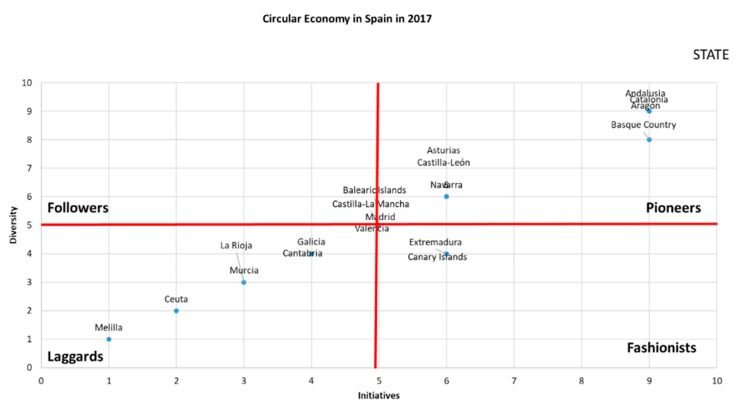
Deployment of the CE in Spain in 2017. Source: own elaboration.

**Figure 4:**
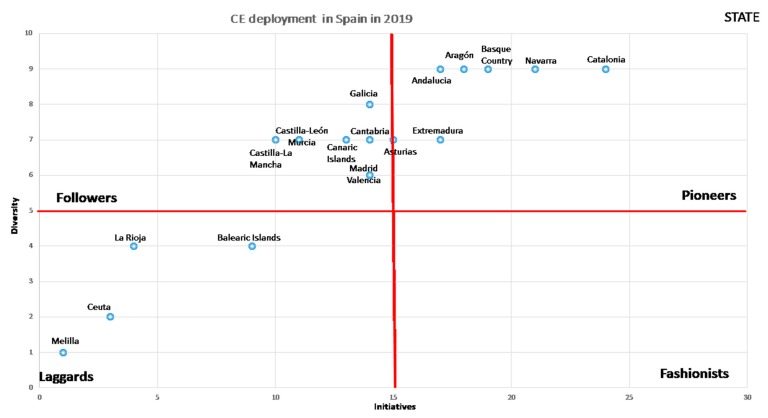
Deployment of the CE in Spain in 2019. Source: own elaboration.

Applying the aforementioned matrix, Figure 3 and Figure 4 show the CE adoption in 2017 and 2019. Thus, CE deployment in the regional territories at the beginning of 2017 is shown in Figure 3.

As it is possible to see in Figure 3, there are two differentiated groups inside the pioneer position. First, a prominent group of regions that they are very close in CE development to the central government and another group of regions close to the follower dimension. Thus, two different groups are identified in the pioneers—one close to the central government and another close to the followers that is a little further from the central government in both intensity and diversity of initiatives. Just in the middle of the matrix, there is another group of regions, very close to being pioneers too. Therefore, more than 50% of regions are following the steps of the central government. In other words, institutional pressure with coercive and normative pressures is working with most of the regions at the moment.

Nevertheless, there is a group of laggards, represented mainly by smaller regions, with the exception of Galicia. Fashionists are acting in a diversity, trying to develop some initiatives by dimension. They do not need a big effort to convert to pioneers. In 2017, neither the central government nor the regions had a very high development of CE, but institutional pressure worked, and most of the regions pursued CE according to the steps of the central government.

The CE deployment in Spain at the end of 2019 is shown in Figure 4. As it is possible to see, hard and soft regulations continue in both the state and regions according to the proposed model (see Table 4).

Several findings can be observed in Figure 4. First, the advance of CE in regions grew in terms of new initiatives on studied period, but less than in the State. This situation has broken the previous pioneer group. As Figure 4 shows, all regions are far from the State. There are two regions, “Catalonia and Navarra”, of the pioneers that are a little closer to state development, while a number of other regions are getting closer and inside the pioneers’ area. Thus, there are two subgroups—Catalonia-Navarra and the rest of regions—inside the pioneers’ area. Therefore, it is possible to assert that the development of new initiatives by regions has been decelerated because the State has accelerated their initiatives deployment.

Second, the followers’ dimension has suffered big changes. On the one hand there have been movements from the position of laggards to followers, but there has also been a regression from pioneers to followers, which reflects mixed results in this area. The followers are a compact and intermediate group of regions inside of followers area.

Third, laggard regions have been reduced and incorporated into the followers’ area. Thus, laggards have been reduced, with only one new region being added to this group—the Balearic Islands.

When we analyze Figure 3 and Figure 4, we can see clear differences with respect to the change that occurred in two years with respect to the initiatives and the dimensions developed for the generation of the CE in the Spanish regions.

In Figure 3, there were few initiatives approved by the regional governments, regarding what the state had developed in 2017. However, in 2018, the regions have come substantially closer to the state legislative development. The regions considered fashionist have also disappeared, while the laggard regions have declined.

This means that a very important part of the regions followed in the wake of the state in the development of the CE. The group of followers changed substantially, both in legislative initiatives and in developed dimensions. As a consequence, it can be affirmed that the greater the state development, the greater the legislative development of the CE in the regions, which will contribute to improve the ratios of the country in its set.

As for the laggards, it can be said that, in just two years, they have decreased and, as can be seen in Figure 4, two of the regions in a short time, with little legislative effort, can change quadrants, which would leave this quadrant with only the two small regions that Spain has in the African continent, which clearly have difficulties regarding both resources and dependencies that are difficult to overcome in the short term.

To develop the mapping, the details of each of the different types of pressure were analyzed and differences were found between them. In analyzing the coercive pressure (Table 7), it cannot be said that this force is explicative in the push of the CE. It seems that all the regions that have a greater legislative intensity are in the pioneers’ quadrant, with the exception of Castilla-La Mancha.

Coercive pressure exists in most of the regions, with a very low development in all of them, except for Castilla-La Mancha as the only one that has three dimensions in green. As can be seen, the dimension of sustainable production is where there is a greater development of hard regulation, while most of the regions fail in the development of a reference or global strategy for the CE territory.

Therefore, it can be considered that the state is playing a fundamental role, marking the path to follow and as a diffuser of the development of the CE. Overall, the development of the CE has been increased in Spain in terms of intensity of initiatives and diversity of topics covered. Thus, these findings show a clear direct institutional pressure followed by state behavior.

However, this CE development could be accelerated by exercising a more active coercive position. This position of strength has advantages and disadvantages. Thus, a certain behavior can be accelerated, but if it is not done with due consensus, attitudes or undesirable behaviors such as greenwashing can be adopted. Therefore, it is necessary to find the right balance between coercive or normative pressures to find the optimal result. Specifically, in the case of Spain, it seems that this optimal position has been found and that the role of the state is pushing the regions into a harmonious adoption of the CE.

Regarding normative pressure, the results show (see Table 8) that regions are not following the State on diffusion. In fact, regulations on different dimensions are scarce, even between the regions that are most advanced in the deployment of the CE.

Therefore, Proposition 1 is accepted in that coercive pressure has a high influence on the CE impulse; however, Proposition 2 can only be weakly accepted, since some influence of normative pressure not associated with a high pressure is detected.

Finally, with regard to mimetic pressure, the aforementioned factors shown in Table 9 will be used to analyze the existence or non-existence of a mimetic behavior between regions. Thus, information has been collected on the population, the size of the region, its location, the weight of the sectors by region, and the contribution of its GDP to global GDP.

**Figure 5:**
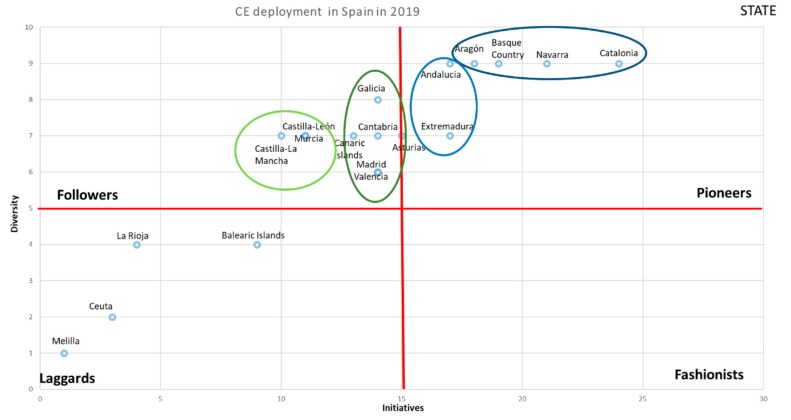
Map with regions grouped by matrix. Source: Own elaboration.

In order to measure whether the existence of mimetic pressure occurs, a comparison is made with these criteria as a basis for the regions found in each of the quadrants in the matrix of 2019, with the aim of finding elements of identification among all or part of the regions (see Figure 5). In the pioneers’ quadrant, two groups have been identified. One in the north and the other in the south. The followers’ quadrant also contains two groups and, finally, the only group of laggards.

**Figure 6:**
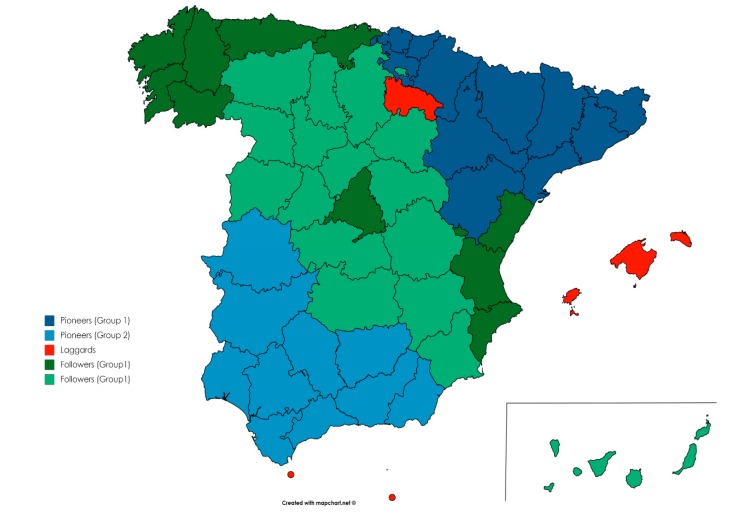
Map with regions grouped by matrix. Source: Own elaboration with https://mapchart.net/spain.html.

Figure 6 grouped regions according to matrix by quadrant in the whole territory. Thus, the pioneers groups are one in the northeast composed by Catalonia, Navarra, Basque Country, and Aragon and another composed by Andalusia and Extremadura—south and southwest. In the followers’ quadrant, there is a group with some dispersion, with regions in the northwest, center, east and southwest, and another group in the center of the country. This latter group is also similar in population, GDP, and in the sectors of associated activity.

Regarding the pioneers’ group, a mimetic behavior is identified among the two groups of regions by geographical proximity. The two group of pioneers are close and have a certain degree of similarity in economic activity. Therefore, in this group we can affirm that a certain level of mimetic behavior could occur.

In the followers’ group we find a certain level of identification between the two pairs of regions—Castilla-León and Castilla-La Mancha and Galicia, Cantabria, and Asturias—that is found in this quadrant, both in terms of population, geographical proximity, and territory and in the sectors of associated activity. Therefore, mimetic pressure could be working between them. The same would happen in the laggards quadrant with the Ceuta and Melilla pair but not with the other laggards. Therefore, it can be concluded that mimetic pressure is occurring in some regions. Therefore, Proposition 3 is partially accepted.

## 7. Conclusions

A number of conclusions can be emphasized. Since the European Commission in 2011 launched a series of communications addressed to the European Parliament, the Council, the European Economic and Social Committee, and the Committee of the Regions, with the title “Roadmap to a Resource Efficient Europe,” it has not stopped encouraging and proposing that the member states take steps toward achieving the definitive implementation of a CE in their territories. All these actions can be considered as coercive pressure. While the 2011 communication proposes a new path to action on the efficiency of resources, with a process involving all key stakeholders, to discuss and agree on indicators and objectives for the end of 2013, the 2015 communication encourages the member states to act consistently with the EU to achieve the global commitments set out in the 2030 Agenda for Sustainable Development. The 2016 communication describes the initiatives that the EU is implementing to achieve the SDGs. In addition, it has been approving directives of obligatory fulfillment on the part of these states, oriented in this same line, becoming normative pressure.

All these actions have meant that, in recent years, the countries that make up the EU have improved in all CE indicators defined by Eurostat: (1) Production and consumption—EU self-sufficiency for raw materials, green public procurement, waste generation, and food waste; (2) waste management—recycling rates for specific waste streams; (3) secondary raw materials—contribution of recycled materials to raw material demand and trade in recyclable raw materials; and (4) competitiveness and innovation—private investments, jobs, gross value added, and patents. This seems to indicate that, thanks to the institutional effort, there has been an improvement, in some cases important and in others lighter, of the indicators for the implementation of a CE model.

This fact fits into institutional theory, which defends institutional pressure having a significant impact by three lines of action: Coercive pressure and normative pressure from government supranationalists and nationalists and mimetic pressure from other countries or regions. This pressure encourages the adoption of initiatives and measures aimed at achieving the effective implementation of a CE in the EU and its member states in an assumable time.

In the specific case of Spain, from 2017, there is an increasing tendency to legislate, both nationally and regionally, promoting and facilitating the transformation of its linear economy into a more CE. While in 2017, 16 legislative initiatives related to the four dimensions of CE collected in the monitoring framework were approved in Spain, in 2019, this figure was increased to 34. However, this legislative and regulatory effort has not been taken on by the different Spanish regions with the same intensity. This has allowed it to achieve an outstanding position among the countries with its environment; in particular, it occupies the third position of the Mediterranean European Union, after Italy and Slovenia, standing out with its considerable percentage of recycled overall packaging, with its reduced generation of municipal waste, and in the important recycling rate of e-waste and overall packaging; hence the importance of institutional engagement at the macro level to push the CE. In Spain, when analyzing the intensity and diversity of its legislative and normative initiatives, significant differences are found. Therefore, the role of coercive pressure is functioning but not normative pressure. This situation could be slowing down the implementation of the CE, but it could also make the CE as consolidating and incorporating in all the dimensions at a speed that allows to consolidate the CE at the macro and meso levels.

It is clear that a certain degree of coerciveness is necessary to lay the foundation for any economic and social change. However, coercive pressure is the most important to boost the CE, in the case of Spain. Coercive pressure contributes to boosting the CE, although it is recommended to strengthen the development of specific coercive tools in the form of laws, sanctions, or aid for the implementation of the CE. Llach et al. [27] state that institutional support at the highest level can be a source of knowledge, resolution of doubts, and funding for the regions.

Mimetic pressure is working to some extent, although it will be necessary to deepen this in the near future. In addition, this study should be carried out for the EU as a whole. This type of pressure seems to be working due to trait based by identification with selected traits such as performance or proximity. Therefore, both coercive and mimetic pressures are driving the CE in Spain.

Therefore, the government should continue to promote the development of the CE through both types of coercive pressure—laws in those dimensions of less development and putting the mechanisms for regulatory development into those dimensions that are more advanced—to achieve the desired levels. In this way, higher level institutions support lower level institutions, especially those with fewer resources, in order to follow the level determined by the state and, in this way, do not compromise the global objectives of adoption. Thus, this situation is positive for the CE’s national progress.

These results could be extrapolated internationally to other Mediterranean countries with similar economic characteristics to Spain, for example, relative to the importance of tourism on its economy. However, most countries do not have a fragmented territorial organization as in the case of Spain, which has 19 regions with a high degree of political autonomy. This is a strong limitation in extending the achieved results to other countries.

Finally, there are also some limitations, which would be resolved with the extension of this study. The modeling of mimetic pressure could also be reinforced. As a future line of research, it is proposed to extend the study to all European countries or countries with a similar internal organizational structure.

## Figures and Tables

**Table 1:** The Development of the Circular Economy (CE) in the European Union (EU).

Title of the Initiative	Type of Initiative	Publication Date	Goals	Link
Roadmap to a resource efficient Europe	Communication from the Commission to the European Parliament, the Council, the European Economic and Social Committee and the Committee of the Regions	20/09/2011	Provide a stable perspective for transforming the economy in the EU	https://eur-lex.europa.eu/legal-content/ES/TXT/PDF/?uri=CELEX:52011DC0571&from=EN
Closing the loop. An EU action plan for the circular economy	Communication from the Commission to the European Parliament, the Council, the European Economic and Social Committee and the Committee of the Regions	02/12/2015	Encourage member states to fulfill the commitments of the 2030 Agenda	https://eur-lex.europa.eu/resource.html?uri=cellar:8a8ef5e8-99a0-11e5-b3b7-01aa75ed71a1.0012.02/DOC_1&format=PDF
Next steps for a sustainable European future: European action for sustainability	Communication from the Commission to the European Parliament, the Council, the European Economic and Social Committee and the Committee of the Regions	22/11/2016	Confirm the EU’s commitment to sustainable development and the achievement of the 17 Sustainable Development Goals (SDGs), describing the initiatives that the EU countries are implementing to reach the 17 SDGs	https://eur-lex.europa.eu/legal-content/EN/TXT/PDF/?uri=CELEX:52016DC0739&from=EN
Key European action supporting the 2030 Agenda and the Sustainable Development Goals	Commission Staff Working Document. Accompanying the Document Next Steps for a Sustainable European Future: European Union Action for Sustainability			https://eur-lex.europa.eu/legal-content/EN/TXT/PDF/?uri=CELEX:52016SC0390&from=en
Monitoring framework for the circular economy	Communication from the Commission to the European Parliament, the Council, the European Economic and Social Committee and the Committee of the Regions	16/01/2018	Provides an analysis of how Member States are implementing the measures aimed at implementing a CE in the EU and what results are being achieved	https://eur-lex.europa.eu/legal-content/EN/TXT/PDF/?uri=CELEX:52018DC0029&from=EN
A European strategy for plastics in a circular economy	Communication from the Commission to the European Parliament, the Council, the European Economic and Social Committee and the Committee of the Regions	16/01/2018	Achieve a circular economy of plastic, promote the reuse of water, encourage sustainable food systems, and reduce food waste	https://eur-lex.europa.eu/resource.html?uri=cellar:2df5d1d2-fac7-11e7-b8f5-01aa75ed71a1.0001.02/DOC_1&format=PDF
A European strategy for plastics in a circular economy	Commission staff working document. Accompanying the document a European strategy for plastics in a circular economy			https://eur-lex.europa.eu/legal-content/EN/TXT/PDF/?uri=CELEX:52018SC0016&from=EN
Directive (UE) 2018/849	Directive of the European Parliament and of the Council amending Directives 2000/53/EC on end-of-life vehicles, 2006/66/EC on batteries and accumulators and waste batteries and accumulators, and 2012/19/EU on waste electrical and electronic equipment	30/05/2018	Update previous directives on vehicles at the end of their useful life, on batteries and accumulators and their waste, and, finally, on waste electrical and electronic equipment	https://eur-lex.europa.eu/legal-content/ES/TXT/?qid=1555698440185&uri=CELEX:32018L0849
Directive (UE) 2018/850	Directive of the European Parliament and of the Council amending Directive 1999/31/EC on the landfill of waste	30/05/2018	Update the previous directive on landfills of waste	https://eur-lex.europa.eu/legal-content/ES/TXT/?qid=1555698440185&uri=CELEX:32018L0850
Directive (UE) 2018/851	Directive of the European Parliament and of the Council amending Directive 2008/98/EC on waste	30/05/2018	Update the previous directive on waste	https://eur-lex.europa.eu/legal-content/ES/TXT/?qid=1555698440185&uri=CELEX:32018L0851
Directive (UE) 2018/852	Directive of the European Parliament and of the Council amending Directive 94/62/EC on packaging and packaging waste	30/05/2018	Update the previous directive on packaging and packaging waste	https://eur-lex.europa.eu/legal-content/ES/TXT/?qid=1555698440185&uri=CELEX:32018L0852
A sustainable bioeconomy for Europe: Strengthening the connection between economy, society and the environment	Communication from the Commission to the European Parliament, the Council, the European Economic and Social Committee and the Committee of the Regions	11/10/2018	Update the bioeconomy strategy of 2012, setting 14 specific actions	https://eur-lex.europa.eu/legal-content/EN/TXT/PDF/?uri=CELEX:52018DC0673&from=EN
Towards a sustainable Europe by 2030	Reflection paper	30/01/2019	The questions raised in this reflection paper are intended to inform a debate with a view to inspiring the debate on the future of Europe, the preparation of the European Union’s strategic agenda 2019–2024, and the priority setting of the next European Commission	https://ec.europa.eu/commission/sites/beta-political/files/rp_sustainable_europe_30-01_en_web.pdf
The implementation of the circular economy action plan	Report from the Commission to the European Parliament, the Council, the European Economic and Social Committee and the Committee of the Regions	04/03/2019	This report presents the main results of implementing the circular economy action plan	https://eur-lex.europa.eu/legal-content/EN/TXT/PDF/?uri=CELEX:52019DC0190&from=ES
Environmental implementation review 2019: A Europe that protects its citizens and enhances their quality of life	Communication from the Commission to the European Parliament, the Council, the European Economic and Social Committee and the Committee of the Regions	04/04/2019	The Environmental implementation review identify the causes of implementation gaps and addressing systemic obstacles to environmental integration across policy sectors	https://eur-lex.europa.eu/resource.html?uri=cellar:fcfafdcd-0abf-11ea-8c1f-01aa75ed71a1.0013.02/DOC_1&format=PDF
United in delivering the energy union and climate action. Setting the foundations for a successful clean energy transition	Communication from the Commission to the European Parliament, the Council, the European Economic and Social Committee and the Committee of the Regions	18/06/2019	This communication analyses the draft national energy and climate plans (NECPs) and looks at their aggregated effects in reaching the EU energy union objectives and 2030 targets	https://eur-lex.europa.eu/legal-content/EN/TXT/PDF/?uri=CELEX:52019DC0285&rid=1
The European green deal	Communication from the Commission to the European Parliament, the European Council, the Council, the European Economic and Social Committee and the Committee of the Regions	11/12/2019	The European green deal is a new growth strategy that aims to transform the EU into a fair and prosperous society, with a modern, resource-efficient, and competitive economy where there are no net emissions of greenhouse gases in 2050, and where economic growth is decoupled from resource use	https://eur-lex.europa.eu/resource.html?uri=cellar:b828d165-1c22-11ea-8c1f-01aa75ed71a1.0002.02/DOC_1&format=PDF
Annual sustainable growth strategy 2020	Communication from the Commission to the European Parliament, the Council, the European Central Bank, the European Economic and Social Committee, the Committee of the Regions and the European Investment Bank	17/12/2019	This communication defends that the European green deal puts sustainability in all of its senses: environment, productivity, stability and fairness	https://eur-lex.europa.eu/legal-content/EN/TXT/PDF/?uri=CELEX:52019DC0650&qid=1581257527301&from=EN

Source: Own elaboration, February 2020.

**Table 2:** Monitoring framework evolution for the CE in the EU (28)**.**

	Year	EU	Year	EU
Production and Consumption				
EU self-sufficiency for raw materials (percentage)	2016	36.4	N/A	N/A
Generation of municipal waste (kg per capita)	2018	488	2011	498
Food waste (million ton)	2016	80	2012	81
Waste Management				
Recycling rate of municipal waste (percentage)	2018	47	2011	39.6
Recycling rate of overall packaging (percentage)	2017	67	2012	64.7
Recycling rate of e-waste (percentage)	2016	41.4	2011	28.7
Recycling of bio-waste (kg per capita)	2018	83	2011	69
Recovery rate of construction and demolition waste (percentage)	2016	89	2010	78
Secondary Raw Materials				
End-of-life recycling input rates (EOL-RIRs) (percentage)	2016	12.4	N/A	N/A
Circular material use rate (percentage)	2017	11.7	2010	10.8
Competitiveness and Innovation				
Gross investment in tangible goods (percentage of gross domestic product (GDP) at current prices)	2017	0.12	2013	0.11
Number of persons employed (percentage of total employment)	2017	1.69	2012	1.68
Number of patents related to recycling and secondary raw materials (per million inhabitants)	2015	0.70	2009	0.60

Source: Own elaboration based on [31]. Note: N/A: Nor available.

**Table 3:** Best values by EU countries for the CE.

	Country with Best Value	Value	Year
Production and Consumption			
Generation of municipal waste (kg per capita)	Romania	272	2018
Waste Management			
Recycling rate of municipal waste (percentage)	Germany	67.3	2018
Recycling rate of overall packaging (percentage)	Belgium	83.8	2017
Recycling rate of e-waste (percentage)	Croatia	81.3	2017
Recycling of bio-waste (kg per capita)	Austria	187	2018
Recovery rate of construction and demolition waste (percentage)	Luxembourg, The Netherlands, and Malta	100	2016
Secondary Raw Materials			
Circular material use rate (percentage)	The Netherlands	29.9 (e)	2017
Competitiveness and Innovation			
Gross investment in tangible goods (percentage of gross domestic product at current prices)	Latvia	0.35	2017
Number of persons employed (percentage of total employment)	Latvia	2.82	2017
Number of patents related to recycling and secondary raw materials (per million inhabitants)	Luxembourg	3.51	2015

(e) Eurostat estimate; Source: Own elaboration based on [31].

**Table 4:** Monitoring framework for the CE in the EU and Spain.

	Year	EU	SPAIN	
**Production and Consumption**				
Generation of municipal waste (kg per capita)	2018	488	475	**↑**
**Waste Management**				
Recycling rate of municipal waste (percentage)	2018	47	36	**↓**
Recycling rate of overall packaging (percentage)	2017	67	68.5	**↑**
Recycling rate of e-waste (percentage)	2017	41.4	41	**↓**
Recycling of bio-waste (kg per capita)	2018	83	84	**↑**
Recovery rate of construction and demolition waste (percentage)	2016	89	79	**↓**
**Secondary Raw Materials**				
Circular material use rate (percentage)	2017	11.7	7.4	**↓**
**Competitiveness and Innovation**				
Gross investment in tangible goods (percentage of gross domestic product (GDP) at current prices)	2017	0.12	0.10	**↓**
Number of persons employed (percentage of total employment)	2017	1.69	2.04	**↑**
Number of patents related to recycling and secondary raw materials (per million inhabitants)	2015	0.70	0.43	**↓**

Source: Own elaboration based on [31]. Note: **↑** means that Spain is better, and **↓** means that Spain is worse.

**Table 5:** Monitoring framework for the CE in the Mediterranean countries.

	Year	UE	Croatia	Place	Cyprus	Place	France	Place	Greece	Place	Italy	Place	Malta	Place	Portugal	Place	Slovenia	Place	Spain	Place
Production and Consumption																				
Generation of municipal waste (kg. per capita)	2018	488	432	1	637	9	527	7	504	6	499	4	604	8	508	6	486	3	475	2
Waste Management																				
Recycling rate of municipal waste (percentage)	2018	47	25.3	6	16.1	8	44	3	18.9	7	49.8	2	6.5	9	28.9	5	58.9	1	36	4
Recycling rate of overall packaging (percentage)	2017	67	50.5	8	64.6	6	68.1	4	68.6	2	66.9	5	39.7	9	55.3	7	70.1	1	68.5	3
Recycling rate of e-waste (percentage)	2017	41.4	81.3	1	23.1	8	36.6	4	32.9	7	34.4	5	10.3	9	43.5	2	33.9	6	41	3
Recycling of bio-waste (kg per capita)	2018	83	12	7	12	7	100	2	21	6	105	1	0	9	85	3	79	45	84	4
Recovery rate of construction and demolition waste (percentage)	2016	89	76	7	57	9	71	8	88	5	98	2	100	1	97	4	98	2	79	6
Secondary Raw Materials																				
Circular material use rate (percentage)	2017	11.7	5.1	6	2.2	8	18.6	1	2.4	7	17.7	2	6.7	5	1.8	9	8.5	3	7.4	4
Competitiveness and Innovation																				
Gross investment in tangible goods (percentage of gross domestic product at current prices)	2017	0.12	0.12	1	0.12	1	0.11	4	0.05	7	0.09	6	nd	8	0.12	1	nd	8	0.10	5
Number of persons employed (percentage of total employment)	2017	1.69	2.21	1	1.99	5	1.64	7	1.52	8	2.06	2	nd	9	1.84	6	2.06	2	2.04	4
Number of patents related to recycling and secondary raw materials (per million inhabitants)	2015	0.70	0	8	1.77	2	0.55	3	0.09	7	0.31	6	2.25	1	0.48	4	0	8	0.43	5
Points				46		63		43		61		35		68		47		39		40

Source: Own elaboration based on [31].

**Table 6:** Legislative impellers of the CE in Spain developed during years 2017 to 2019.

Dimension Monitoring Framework	Legislative Drivers	Number Initiatives 2017	Number Initiatives 2018	Number Initiatives 2019	Total Initiatives	Hard Regulation	Soft Regulation
(1) Production and consumption	-Food policy and CE	2	0	0	2	0	2
	-Sustainable production	3	3	5	11	3	8
	-Sustainable consumption	1	2	1	2	2	0
Total		6	3	6	15	5	10
(2) Waste management	-Waste management policy	4	2	4	10	1	9
	-Plastics policy	0	0	2	2	0	2
	-Waste management especial policies	3	0	0	3	1	2
Total		7	2	6	15	2	13
(3) Secondary raw materials	-Eco-design	1	0	0	1	0	1
	-Biomass policy	4	0	1	5	0	5
Total		5	0	1	6	0	6
(4) Competitiveness and innovation	-Research, development and innovation (R+D+i)	2	1	1	4	0	4
	-Circular Economy Strategy	1 (ongoing)	0	0	1	0	1 (ongoing)
Total		3	1	1	5	0	5
Total initiatives		21	6	14	41	7	34

Source: Own elaboration. Research data compilation to 2020, January.

**Table 7:** Mapping of CE coercive pressure in Regions.

	EC Strategy	Eco-Design	Sustainable Production	Sustainable Consumption	Waste	R+D+i	Plastic Policy	Food Policy	Waste Management Special Policy	Biomass
**Andalusía**										
**Aragon**										
**Asturias**										
**Cantabria**										
**Castilla La Mancha**										
**Castilla-Leon**										
**Catalonia**										
**Ceuta**										
**Community of Madrid**										
**Valencian Community**										
**Estremadura**										
**Galicia**										
**Balearic Islands**										
**Canary Islands**										
**Community of La Rioja**										
**Melilla**										
**Foral Community of Navarre**										
**Basque Country**										
**Muccia**										

Hard regulation: Green = above average; orange = average; red = below average.

**Table 8:** Mapping of CE normative pressure in regions.

	EC Strategy	Eco-design	Sustainable Production	Sustainable consumption	Waste	R+D+i	Plastic Policy	Food Policy	Waste Management Special Policy	Biomass
**Andalusía**										
**Aragon**										
**Asturias**										
**Cantabria**										
**Castilla La Mancha**										
**Castilla-Leon**										
**Catalonia**										
**Ceuta**										
**Community of Madrid**										
**Valencian Community**										
**Estremadura**										
**Galicia**										
**Balearic Islands**										
**Canary Islands**										
**Community of La Rioja**										
**Melilla**										
**Foral Community of Navarre**										
**Basque Country**										
**Muccia**										

Soft regulation: Green = above average; orange = average; red = below average.

**Table 9:** Main characteristics of the regions to measure mimetic pressure.

	Population (1)	Gross Domestic Product (2)		Location/Surface (km^2^) (3)	
		(€)	%		
Spain	46,722,980	1,207,463,136	100%	-	505,990
Andalusia	8,384,408	160,811,516	13.3%	South	87,599
Aragon	1,308,728	37,691,459	3.1%	Northeast	47,720
Asturias	1,028,244	23,650,195	2.0%	Northwest	10,604
Balearic Islands	1,128,908	31,490,768	2.6%	East	4992
Canary Islands	2,127,685	46,029,185	3.8%	Southwest	7447
Cantabria	580,229	13,837,621	1.1%	North	5321
Castilla-La Mancha	2,026,807	41,926,427	3.5%	Center	79,461
Castilla- León	2,409,164	58,816,818	4.9%	Center	94,224
Catalonia	7,600,065	231,277,107	19.2%	Northeast	32,113
Valencian Community	4,963,703	112,127,515	9.3%	East	23,255
Extremadura	1,072,863	19,396,733	1.6%	West	41,634
Galicia	2,701,743	62,878,404	5.2%	Northwest	29,575
Community of La Rioja	315,675	8,391,237	0.7%	Center	5045
Community of Madrid	6,578,079	230,018,098	19.0%	Center	8028
Murcia	1,478,509	31,258,596	2.6%	Southeast	11,300
Foral Community of Navarra	647,554	20,554,871	1.7%	North	10,391
Basque Country	2,199,088	74,040,758	6.1%	North	7234
Ceuta	85,144	1,700,982	0.1%	South	20
Melilla	86,384	1,564,846	0.1%	South	12

Sources: (1) https://www.ine.es/jaxiT3/Datos.htm?t=2915. Accessed, June 28, 2019; (2) Spanish Regional Accountability. Available in www.ine.es. Accessed, June 28, 2019. (3) https://www.ine.es/jaxiT3/Datos.htm?t=2915. Accessed, June 28, 2019.
